# Cyclic AMP Signaling in Biliary Proliferation: A Possible Target for Cholangiocarcinoma Treatment?

**DOI:** 10.3390/cells10071692

**Published:** 2021-07-04

**Authors:** Leonardo Baiocchi, Ilaria Lenci, Martina Milana, Lindsey Kennedy, Keisaku Sato, Wenjun Zhang, Burcin Ekser, Ludovica Ceci, Vik Meadows, Shannon Glaser, Gianfranco Alpini, Heather Francis

**Affiliations:** 1 Hepatology Unit, University of Tor Vergata, 00133 Rome, Italy; baiox@hotmail.it (L.B.); ilaria.lenci@uniroma2.it (I.L.); martinamilana@gmail.com (M.M.); 2 Hepatology and Gastroenterology, Medicine, Indiana University, Indianapolis, IN 46202, USA; linkenn@iu.edu (L.K.); keisato@iu.edu (K.S.); lceci@iu.edu (L.C.); vikmead@iu.edu (V.M.); 3 Richard L. Roudebush VA Medical Center, Indianapolis, IN 46202, USA; 4 Division of Transplant Surgery, Department of Surgery, Indiana University, Indianapolis, IN 46202, USA; wenzhang@iu.edu (W.Z.); bekser@iupui.edu (B.E.); 5 Department of Medical Physiology, Texas A&M University College of Medicine, Bryan, TX 77807, USA; sglaser@tamu.edu

**Keywords:** cholangiocarcinoma, cAMP, cholangiocytes, proliferation, PKA, secretin

## Abstract

Cholangiocarcinoma is a lethal disease with scarce response to current systemic therapy. The rare occurrence and large heterogeneity of this cancer, together with poor knowledge of its molecular mechanisms, are elements contributing to the difficulties in finding an appropriate cure. Cholangiocytes (and their cellular precursors) are considered the liver component giving rise to cholangiocarcinoma. These cells respond to several hormones, neuropeptides and molecular stimuli employing the cAMP/PKA system for the translation of messages in the intracellular space. For instance, in physiological conditions, stimulation of the secretin receptor determines an increase of intracellular levels of cAMP, thus activating a series of molecular events, finally determining in bicarbonate-enriched choleresis. However, activation of the same receptor during cholangiocytes’ injury promotes cellular growth again, using cAMP as the second messenger. Since several scientific pieces of evidence link cAMP signaling system to cholangiocytes’ proliferation, the possible changes of this pathway during cancer growth also seem relevant. In this review, we summarize the current findings regarding the cAMP pathway and its role in biliary normal and neoplastic cell proliferation. Perspectives for targeting the cAMP machinery in cholangiocarcinoma therapy are also discussed.

## 1. Introduction

**Figure 1:**
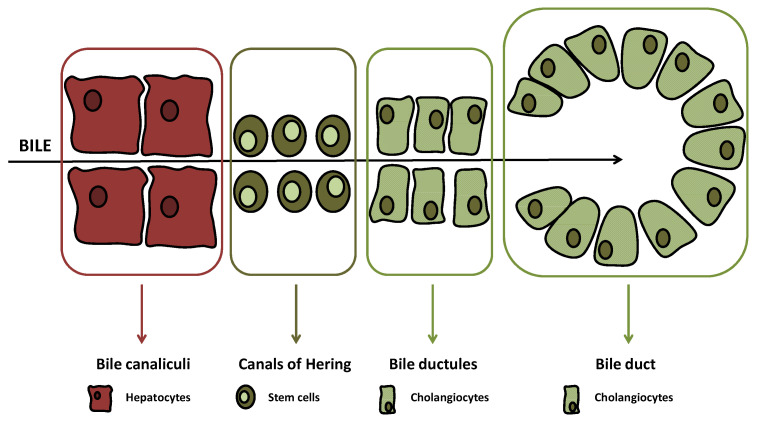
The main cells and ultrastructural districts encountered by the bile (black arrow) while moving from the hepatocytes to the bile ducts.

The biliary tree is lined by epithelial cells (i.e., cholangiocytes), which appear to originate from a common stem cell compartment, the hepatic progenitor cells, similar to hepatocytes [1]. These pluripotent cells are located at the interface between the hepatocyte canaliculi and bile ductules in the canals of Hering (Figure 1), the latter being the smaller branches of the biliary tree [2].

The canals of Hering extend to the interlobular ducts (i.e., bile ductules, 15–100 μm in diameter), then continue in the septal area, segmental and finally, in the right and left hepatic ducts, joining together at the hepatic hilum. This portion of the biliary epithelium is regarded as the intrahepatic one, whereas the part extending outside the liver and comprising the common bile duct, the gallbladder (with cystic duct) and choledochus is defined as extrahepatic [1]. Via electron microscopy, two different cholangiocyte subpopulations in rodent livers are well recognized (small and large), lining small ducts (≤15 μm in diameter) or large ducts (≥15 μm in diameter) according to their size [1,3]. Large cyclic adenosine monophosphate (cAMP)-dependent cholangiocytes are considered the functional part of the biliary tree since they respond to gastrointestinal hormones (e.g., secretin), neuro-peptides and other modulators [4,5,6]. On the other hand, small Ca^2+^-dependent cholangiocytes [7] do not respond to secretin and proliferate to replenish the biliary epithelium (during damage to the large, cAMP-dependent cholangiocytes) by amplification of Ca^2+^-dependent signaling and *de novo* acquisition of large cholangiocyte phenotypes [6,8,9]. While, at the beginning, just bile duct secretory activities were studied with regard to biliary tract diseases, lately also, biliary proliferative phenotypes have gained interest [10,11,12]. For instance, atypical cholangiocyte proliferation, characterized by truncated tortuous bile ducts, has been observed in adult cholestatic liver diseases such as primary biliary cholangitis (PBC) and primary sclerosing cholangitis (PSC) [13,14]. Both diseases are defined as ductopenia, since changes and evolution of proliferation determine a reduction of total bile ducts in the end [15].

In cholangiocarcinoma (CCA), cellular growth and expansion also deserve interest as possible targets for therapy. Several molecular cascades such as the Janus kinase/signal transducer and activator of transcription, p38 MAP kinase (MAPK), Akt (AKA, protein kinase B, PKB) or fibroblast growth factor/fibroblast growth factor receptor may stimulate cell growth and impact biliary proliferation [16,17]. Both hyperplastic and neoplastic cholangiocyte growth are frequently associated with changes in intracellular cAMP levels [18]. In this review, after a general description of cAMP signaling and its role in parenchymal and biliary cell functions, we will discuss the most recent findings regarding the relationship between the cAMP-dependent pathway and cancer of the biliary tract (e.g., cholangiocarcinoma).

## 2. cAMP Signaling

**Figure 2:**
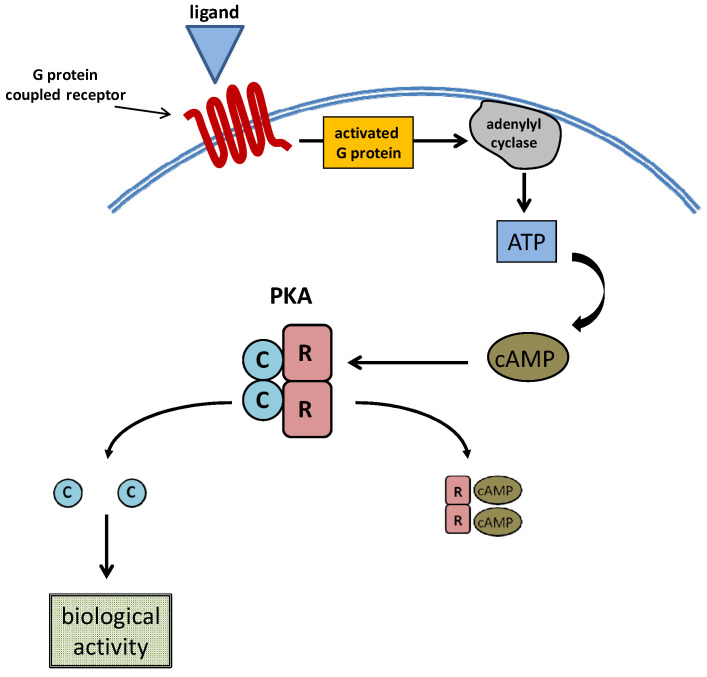
The typical mechanism of PKA activation upon stimulation of G-protein-coupled receptor. Ligand-receptor binding determines an increase of intracellular cAMP. This, in turn, attaches to the reactive portions of PKA (R), allowing the release of the catalytic subunits (C) responsible for the following biological effects.

cAMP was first discovered in 1958 and introduced the concept of a “second messenger” system [19]. In fact, this molecule, together with cyclic guanosine monophosphate (cGMP), has been identified as an important intracellular translator of membrane signaling originating from hormones, growth factors, cytokines and other molecules [20]. In the general transduction mechanism, the stimulated receptor activates the corresponding G-coupled protein, leading to increased adenylyl cyclase-mediated cAMP synthesis. The following steps include cAMP binding to protein kinase A (PKA) or an exchange protein activated by cAMP (EPAC) and also cyclic nucleotide-gated ion channels [21]. Canonical G-protein-coupled receptor/cAMP/PKA signaling is depicted in Figure 2.

cAMP in eukaryotic cells serves as a secondary messenger for several biological activities such as the growth, differentiation and expression of selected mRNAs [22]. As described before, cAMP is primarily synthesized by G-protein-dependent stimulation of adenyl cyclase; however, G-protein-independent synthesis of cAMP may also occur upon stimulation of a soluble adenylyl cyclase induced by bicarbonate [23]; its activity is interrupted by phosphodiesterase-mediated degradation, when the biological target is achieved. The activity of PKA is prompted by cAMP binding to the R subunit that allows detachment of the C catalytic portion [24]; PKA is tetrameric so it is composed of two R and two C portions. Two different PKA isoforms (I and II) may be distinguished on the basis of different R subunits (I and II), while the C portion remains unchanged. At the same time, both the RI and RII subunits may present two forms called α and β, respectively [25]. Heterogeneity in the composition of subunits, and its different distribution in cellular districts is the basis for the several effects that PKA may exert upon stimulation [26]. Several cytoplasmic and nuclear proteins are targeted by PKA [20]. On the other hand, significant gene transcription activity is associated with PKA-dependent stimulation of the cAMP response element-binding protein (CREB) [27]. While PKA was considered the unique cAMP target for several years, recently, EPAC 1 and 2 were identified as sharing an identical PKA-subunit R-binding domain [28]. EPACs also regulate several physiological mechanisms including cellular secretion, adhesion and differentiation [22]. Finally, the cAMP binding domain has been identified at cyclic nucleotide-gated ion channels; cAMP-mediated channels’ stimulation remains of paramount importance for neuronal impulse transmission and cardiomyocyte contractility [29].

## 3. cAMP Signaling in Hepatocytes

### 3.1. General Signaling Effects

Similar to what is observed in other organs, in the liver, cAMP signaling represents an important point of intersection for signaling activities both in normal and diseased conditions. In this section, we discuss the main activities supported by the cAMP transduction pathway machinery in hepatocytes. The cAMP/PKA axis is involved in both liver gluconeogenesis and glycogenolysis [30]. In fact, during starvation, glucagon stimulates cAMP/PKA/CREB signaling. The maximal stimulation for transcription of target glucogenic genes is acquired by binding CREB, together with its CREB-regulated transcription coactivator 2 (CRTC2) [31]; conversely, glycogenolysis may be stimulated by PKA, leading to enhanced activity of glycogen phosphorylase. Nonetheless, fat metabolism may be regulated in the liver by cAMP levels. In fact, cAMP-stimulated PKA intracellular levels may phosphorylate and inhibit key enzymes of lipogenesis [32]. Evidence supporting the role of cAMP signaling in glucose and fat homeostasis has stimulated studies on liver diseases characterized by metabolic derangement, such as alcoholic liver disease (ALD) or non-alcoholic fatty liver disease (NAFLD).

### 3.2. cAMP in Liver Diseases with Metabolic Impairment

Reduced levels of cAMP have been shown in peripheral mononuclear cells from ALD patients in the early eighties [33]. Recently, decreased activity of cytochrome P450 2E1 (CYP2E1, the main enzyme producing toxic acetaldehyde from ethanol) by cAMP, was demonstrated in an experimental rodent model of ethanol feeding [34]. Similarly, in an analog animal model, a study demonstrated that ethanol-related phosphodiesterase-4 stimulation induced a decrease in intracellular cAMP levels and subsequent fat accumulation within the liver [35]. Beside the effects on lipid homeostasis, cAMP may also exhibit anti-inflammatory properties in ALD. In fact, a study in isolated monocytes and rat Kupffer cells demonstrated decreased cAMP levels associated with increased release of tumor necrosis factor-α (TNF-α) after alcohol exposure [36]. Furthermore, cAMP has been shown to attenuate alcohol-induced oxidative damage stimulating nitric oxide synthase expression [37]. With regards to NAFLD, a study has shown that cAMP acts as a second messenger after glucagon-like peptide (GLP) 1 receptor stimulation, improving fatty liver accumulation, glucose homeostasis and liver serum chemistry in leptin-deficient (*ob/ob*) mice [38]. Recent findings suggest that, together with peroxisome proliferator-activated receptor alpha (PPAR-α) and forkhead box O1 (FOXO1), the cAMP/responsive element-binding protein H (CREBH) signaling pathways remain of paramount importance in metabolic liver diseases [39]. Although a conclusive model describing the complex relationship between the cAMP/CREBH pathway and NAFLD (also for the involvement of this pathway in glucose homeostasis, the stress response and the inflammatory process) is lacking, this signaling system may be a good therapeutic target for the human metabolic liver diseases [40]. Finally, the role of cAMP in hepatocyte proliferation is well-defined [41]. In the model of 70% partial hepatectomy, which represents an ideal system to evaluate cellular regeneration, a two-phase increase in cAMP and activation of different PKA subtypes is observed. Therefore, important CREB phosphorylation is reported, thus stimulating the downstream growth genes; this process requires a functional G-protein, Gαs [42].

## 4. cAMP Signaling in Cholangiocytes

### 4.1. cAMP and Biliary Secretion

Since the beginning of research on biliary epithelia, cAMP attracted attention for its involvement in secretin-stimulated biliary secretion [4]. In fact, several studies established the role of secretin in the stimulation of biliary bicarbonate secretion by specifically increasing cAMP intracellular levels in cholangiocytes, but not hepatocytes [4,43,44]. In a seminal study, secretin-stimulated exocytosis was examined in detail in isolated cholangiocytes [44]; the study demonstrated that exocytosis was closely associated with enhanced intracellular cAMP levels (more than 200% increase compared to unstimulated cholangiocytes), independent of cGMP intracellular levels, as well as stimulated by forskolin (a drug enhancing cAMP synthesis). Following interaction with its basolateral receptors [4,5,45], secretin-stimulated choleresis is mediated by sequential activation of PKA, the cystic fibrosis transmembrane conductance regulator (CFTR) and anion exchanger 2 (AE2), leading to enhanced bicarbonate secretion [4,5,46,47]. In the bile duct-ligated (BDL) hyperplastic rodent model, this event is amplified [47] but is inhibited by insulin, decreasing cAMP intracellular levels through a Ca^2+^/PKC-dependent mechanism [48].

### 4.2. cAMP and Biliary Proliferation

A number of studies also demonstrated the effects of cAMP signaling on biliary proliferation in response to liver injury [10,49,50,51]. Recently, research specifically focused on the net effect of cAMP on proliferation of biliary epithelia was performed [49]. Administration of forskolin (an activator of adenylyl cyclase) to Fisher male rats significantly increased intrahepatic biliary mass, as well as biliary secretion. Also, the signaling pathway was characterized in this study, demonstrating the involvement of cAMP in the activation of a signaling cascade composed by the PKA/Src/MEK/ERK1/2 axis [49]. This study clarified some relevant concepts, such as how: (i) cAMP plays an important messenger role for both secretive and proliferative biliary functions; (ii) a functional cAMP/ERK signaling axis is present in cholangiocytes; and (iii) in the biliary ductal system, the cAMP/ERK signaling axis promotes cell growth [10,49]. Another study demonstrated that prolonged secretin administration stimulated cAMP-dependent biliary growth in normal rats [52]. Minipump administration of the hormone (2.5 nmoles/kg BW/day) for one week increased biliary proliferation and hyperplasia by enhancing intracellular cAMP levels. Furthermore, in BDL rats lacking a secretin receptor (knockout SR^−/−^) [53], there was reduced biliary growth through a significant decrease in intracellular cAMP and ERK1/2 phosphorylation. Other studies underscored the important function of cAMP as a common hormonal and neuroendocrine signal mediating cholangiocyte growth [10,49]. With regard to gastrointestinal hormones, while secretin and GLP-1 stimulate biliary proliferation by enhancing cAMP machinery [10,53,54], others such as gastrin and somatostatin reduced biliary hyperplasia. However, while the inhibitory effects of somatostatin on cAMP-dependent biliary proliferation were directly linked to cAMP, gastrin inhibition of cAMP-dependent biliary growth was mediated by changes in the Ca^2+^-dependent protein kinase C (PKC) alpha [55,56,57]. The neuropeptides acetylcholine, serotonin, histamine, melatonin and others also modulate cholangiocyte growth, reacting with specific receptors and altering cAMP levels [58,59,60,61].

Finally, bile acids (BAs) are probably the most important non-endocrine regulators of biliary growth. In molecular studies, some BAs G-protein-coupled receptors have been identified in cholangiocytes such as the Takeda G-protein-coupled receptor 5 (TGR5), the subtype 3 muscarinic (M3) receptor and the sphingosine-1-phosphate receptor 2 (S1PR2) [62]. Among these, TGR5 has been found to regulate cell growth, modulate the cAMP pathway and have an opposite effect when comparing non-ciliated cholangiocytes (increases growth) with ciliated ones (decreases growth) [63]. In fact, activation of TGR5 located in the cilia determines receptor coupling with Gα_i_ instead of Gα_s_, thus decreasing cAMP intracellular levels and cell proliferation, a mechanism impaired in polycystic liver disease. With regard to this latter disease, characterized by an exuberant growth of cysts, possible downregulation of cAMP signaling may be hypothesized as a possible therapeutic approach.

Data from in vivo studies have shown that feeding taurocholic or taurolithocholic acid to rats increases biliary proliferation and secretin-stimulated ductal secretion (a functional index of cholangiocyte growth) [10,49,64], phenotypes that were mediated by increased biliary cAMP levels [65]. On the other hand, ursodeoxycholic acid (UDCA), used for therapy in PBC [66], reduced cholangiocyte proliferation in the cholestatic rodent model of bile duct ligation (BDL) through a decrease in cAMP signaling mediated by activation of PKC alpha [67]. Taken together, the findings suggest that modulation of cholangiocyte growth based on the activity of the cAMP axis normally occurs in physiologic conditions; moreover, this signaling pathway may represent an important target for pharmacological modulation of biliary secretive and proliferative activity in cholangiopathies. However, is to be underscored that the final effect of the increase (or decrease) of cAMP in cholangiocytes is variable according to different molecular factors. In fact, this second messenger lies at the intersection between several signaling cascades with possible divergent downstream biological effects.

## 5. cAMP and Cancer

The role of cAMP and its main effector PKA in cancer has been recognized [68], and targeting of this signaling axis has been identified as a possible strategy for cancer therapy [69]. Changes to or modulation of cAMP/PKA signaling exert effects in a number of cancers involving the brain [70,71], lungs [72], prostate [73] or blood [74]. However, it has to be emphasized that stimulation of cAMP signaling may have opposing results depending on the tissue and cancer type. For instance, in tumor spheres obtained from a primary cell culture of medulloblastoma, cancer growth was inhibited by forskolin and enhanced by PKA inhibition [75]. On the other hand, cAMP/PKA signaling was involved in enhanced expression of the androgen receptor and tumor growth in prostate cancer [76]. Since PKA composed of an RI subunit was linked to increased proliferation, and enhanced expression of the RIα-type was observed in tumors, in the early 1990s, a study with an antisense oligonucleotide-repressing RIα was conducted on cancer cells [77]. Neoplastic cell lines from the stomach, colon and breast (TMK-1, LS-174T and MCF-7 lines) all exhibited impaired growth upon antisense treatment [77]. A following study demonstrated that the antisense oligonucleotide GEM231 also exhibited anticancer activity in vivo [77]. Further, a separate study demonstrated that the antisense oligonucleotide GEM231 also exhibited anticancer activity in vivo [78]. In fact, in a nude mouse model of human prostate, colon, lung and pancreas cancer cells’ xenotransplantation, simultaneous GEM231 administration determined enhanced irinotecan antitumor activity (68). In a Phase I study on 20 patients with different solid tumors, GEM231 (220 mg/m^2^, twice a week) was administered and coupled with a variable dose of Docetaxel [79]. Liver toxicity was frequently observed (increase of transaminases levels in 75% of patients) as well as neutropenia (45%) and fatigue (40%). Neurologic side effects occurred in five patients (20%), with one exhibiting toxic neuropathy; three serious adverse events occurred during the study: a cardiac arrest, a pulmonary embolism and fever as a consequence of neutropenia. Results of further attempts of this study have not been published thus far. Other preclinical studies evaluated an approach with cAMP analogs. In this perspective, 8-Cl-cAMP (i.e., tacladesine) with a preferential inhibition of PKAI evidenced a significant effect on the growth of the following cancer cellular lines: HL-60 leukemia cells [80], MCF-7 (breast), LS-174T (colon) and A549 (lung) [81]. Two trials were registered with 8-Cl-cAMP, one (Phase II, NCT00004902) on multiple myeloma and one (Phase I, NCT00021268) on colon cancer [82]. The results of these studies (despite beginning 20 years ago) have not been disclosed so far. Use of phosphodiesterase for the modulation of cAMP has been suggested by some authors for cancer therapy, but preclinical or clinical results employing this approach have not been published [28].

## 6. CCA and cAMP Signaling

### 6.1. CCA

CCA is a rare and lethal disease representing the most frequent primary liver cancer after hepatocellular carcinoma (HCC) [83]. Incidences of CCA change widely among different countries, ranging from 85 to less than 1 out of 100,000, thus reflecting the prevalence of some risk factors such as parasitic infections (*Clonorchis sinensis* and *Opisthorchis viverrini*), biliary tract disorders (PSC, hepatolithiasis, biliary cystic diseases) and also inflammatory bowel diseases [84]. CCA’s clinical classification relies on its anatomical location along the biliary tree. Intrahepatic (iCCA), perihilar (pCCA) and distal (dCCA) forms are recognized with different prevalence and prognoses [85]. Histological classification is complex since heterogeneity is found in genetic alterations, pathogenesis and cellular origin, which are all factors contributing to a variable morphological picture [86]. Of concern, while some forms of CCA (iCCA) increased their global prevalence and associated mortality in the last decades, treatment of this cancer did not show, in parallel, any major improvements [87,88]. Improved understanding of the processes at the origin of CCA proliferation within the biliary tract may probably stimulate targeted therapy for this cancer in the future. In this perspective, since cAMP signaling plays an important role in the normal proliferative activity of cholangiocytes, it is likely that changes to this pathway may occur in biliary tract cancer.

### 6.2. Hormones/Neuropeptides Modulation of cAMP in CCA

**Figure 3:**
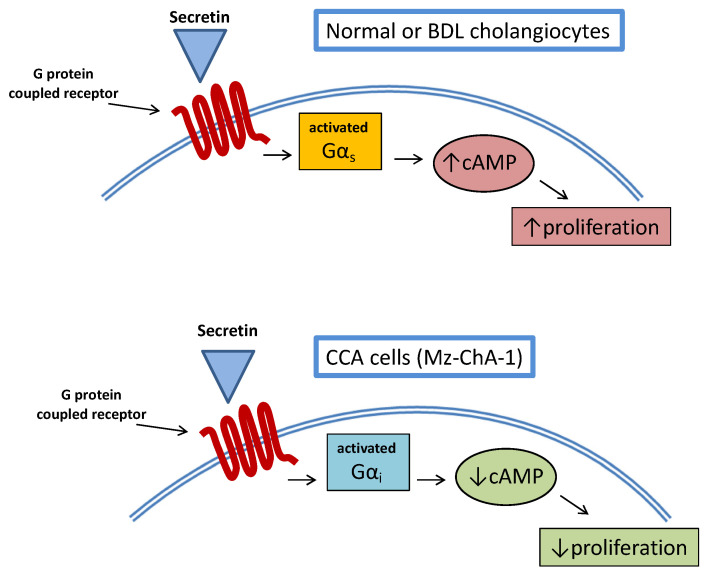
The mechanism and opposing effect of secretin-stimulated G-protein-coupled receptor on normal (or BDL) cholangiocytes and CCA cells (Mz-ChA-1 line).

Secretin-stimulated intracellular cAMP levels represent one of the most studied mechanisms at the origin of cholangiocyte proliferation [89]. A study examined the relationship between cAMP and secretin stimulation in CCA cell lines [90]. Interestingly, the study demonstrated decreased expression of the secretin receptor in the majority of CCA lines (Mz-ChA-1, HuH-28, SG231, CCLP1) used. The same was observed in CCA human liver specimens. More interestingly, stimulation of CCA cell lines with secretin did not increase intracellular cAMP levels so that proliferative activities were not enhanced. This was also confirmed in an in vivo model of Mz-ChA-1 xenotransplantation in nude mice, where tumor growth was delayed by secretin treatment. The opposite effect of secretin on CCA cell growth was related to an aberrant (cancer-related) coupling of the secretin receptor with Gα_i_ rather than Gα_s_ (Figure 3).

This study also had the merit of demonstrating once again the complex interplay between CCA and cAMP signaling, evidencing a mechanism similar to what was already observed, upon stimulation of the TGR5 receptor in cholangiocytes’ cilia [66]. A number of studies suggest a multifaceted interaction between hormones/neuropeptides and cAMP signaling with regard to CCA growth. Somatostatin has been shown to reduce secretin-induced biliary secretion and proliferation, downregulating cAMP intracellular levels in cholangiocytes from BDL rats [56]. There is also evidence that somatostatin inhibits CCA growth [91]. In fact, human CCA specimens and the human CCA line, SK-ChA-1, express a functional somatostatin receptor subtype 2 (SSTR2) [91]. Treatment with a somatostatin analog (SS-14, octreotide) significantly decreased CCA cell growth in CCA lines, whereas lanreotide (a long-acting somatostatin analog) determined a 33% reduced growth of SK-ChA-1 cells implanted in athymic mice. However, this study did not observe any major changes in intracellular cAMP levels, thus excluding a possible role of this signaling system in somatostatin-induced decreased CCA proliferation. On the other hand, a relationship was found between the cAMP system and CCA growth upon stimulation of the α_2_-adrenergic receptor (α_2_-AR) [92]. Mz-ChA-1 and TFK-1 human CCA cell lines expressed α_2_-AR. Exposure of these CCA cells to the α_2_-AR agonist (UK14,304) decreased their growth as demonstrated by reduced [^3^H] thymidine incorporation and PCNA mRNA expression. This effect was related to increased cAMP/PKA signaling activity, inhibiting Raf-1 and B-Raf with a consequent downregulation of MAPK. Another study evaluated the effect of γ-aminobutyric acid (GABA) on CCA growth [93]. Mz-ChA-1, HuH-28 and TFK-1 human CCA cell lines all expressed functional GABA receptors and exposure to GABA decreased the proliferation index and PCNA mRNA expression in all three cell lines. In addition, Mz-ChA-1 cells’ transplantation in nude mice demonstrated reduced tumor growth after 82 days of GABA injection. These effects were related to increased cAMP/PKA signaling activity since: (i) this pathway was enhanced by GABA and; (ii) CCA growth was restored, adding into the cell culture a PKA inhibitor (Rp-cAMP116816). Also, the migration of CCA cells seemed to be impaired by GABA. In fact, wound closure (obtained by linear abrasion to culture plate) required a longer time in GABA-treated cells (>96 h).

### 6.3. BAs Modulation of cAMP in CCA

Other studies examined the possible role of the BA receptor TGR5 (known as a significant stimulator of cAMP/PKA signaling in cholangiocytes) [63] in CCA growth. This receptor has been found to be overexpressed in human CCA specimens when compared to surrounding normal tissue [94,95,96]. Moreover, the TGR5 expression rate was associated with less-differentiated CCA in one study [95]. Stimulation of this receptor in CCA cell cultures (EGI-1 and TFK-1) sustained the proliferation and invasiveness of tumoral cells [94]. TGR5’s stimulating effect on CCA growth seems to occur by a dual mechanism; (i) increased proliferation for stimulation of the EGFR/ERK1/2 pathway; and (ii) inhibition of apoptosis by a cAMP/PKA/CD95 cascade [62]. These observations underscore the differential and complex effect of TGR5/cAMP signaling in cholangiocytes, according to different species and conditions. In fact, while in H69 human biliary cells (both ciliated or non-ciliated), TGR5–induced proliferation is strictly related to fluctuations of cAMP levels [63], in murine cholangiocytes and human CCA cell lines (EGI-1 and TFK-1), cAMP signaling mainly contributes to apoptosis suppression, and growth is maintained by stimulation of the EGFR/ERK1/2 pathway [94]. Further studies, possibly on human tissue, would be needed to improve our knowledge of the TGR5/cAMP relationship in cholangiocytes and possible targeting in CCA.

### 6.4. PKA Subunits Changes in CCA

Changes in PKA subunits have been associated with CCA. Increased expression of RIα was found in human CCA in comparison with the surrounding non-tumoral tissue [97]. In the same study, also in CCA cell lines (M156, OCA17, KKU100 and M214), an enrichment of PKA subunit RIα was observed. Lentiviral transfection-induced knockdown of RIα in M156 and OCA17 cell cultures led to a significant decrease of PKA activity and cellular growth. Reduction of cellular proliferation was in part also related to increased apoptosis. Similar effects were also reported when testing H-89 (a PKA inhibitor) or 8-Cl cAMP on M156, OCA17, KKU100 and M214 cell culture growth. In fact, reduced CCA cellular proliferation, accounting for 20–40% growth inhibition, was observed, according to different doses of the inhibitor or cellular line. Resulting from these findings, an attempt was made to develop T-cell expansion against CCA, employing self-differentiated monocyte-derived dendritic cells presenting an RIα subunit [98]. Effector T-cells, activated with this strategy, were able to eliminate nearly 60% of CCA cells in vitro. PRKACA and PRKACB, the genes involved in transcription of the α and β PKA catalytic subunits, have also been found altered in some cases of CCA [99]. Their gene fusion with ATP1B may determine increased transcription with downstream activation of proliferative signals [100]. Similar fusion (DNAJB1-PRKACA) has been observed in fibrolamellar HCC; however, this genetic aberration should not be considered specific for this latter tumor since it was also identified in pancreatic and biliary tract cancer [101]. Recently, a study focused on intraductal oncocytic papillary neoplasm (IOPN) and PRKACA or PRKACB fusion [102]. Among 23 patients, three were harboring an IOPN of the biliary tract. All of them had ATP1B1–PRKACB gene fusion. The same alterations were not found in other malignancies of the pancreas or biliary tract, suggesting that PRKACA or PRKACB fusion may be characteristic of the IOPN type. The main preclinical studies evaluating the cAMP pathway in CCA are reported in Table 1.

## 7. Conclusions/Future Perspectives

CCA is a severe disease and we are still in search of appropriate treatment. While a limited number of patients may undergo surgical resection with improved survival, the majority of subjects harboring CCA are expected to die within one year of diagnosis [103]. Several efforts have been made in preclinical studies to identify possible strategies for CCA systemic therapy [99]. Targeting genetic aberrations or the use of immune checkpoint inhibitors represent possible opportunities in the future [88]; however, exploring new pathways for therapy seems wise given the suboptimal results of the current medical practice. cAMP/PKA signaling is a highly conserved pathway in eukaryotic cells and its important contribution in parenchymal and non-parenchymal epithelial liver cell growth has been largely demonstrated. Since important changes to this pathway occur in several human cancers, its targeting has been attempted with antisense oligonucleotide or cAMP analogs in preclinical models. Data on CCA when employing this approach are scanty, despite the fact that cAMP/PKA signaling has been demonstrated to be of paramount importance in the regulation of cholangiocyte proliferative activities in physiologic conditions and during damage. Moreover, as cholangiocytes are the main liver collectors of several hormones, neuropeptides and angiogenic factor signals employing cAMP as a second messenger, the possible modulation of this pathway employing these molecules may be hypothesized. In this context, we demonstrated that secretin receptor stimulation in CCA cells is not followed by the physiologic increase of cAMP that supports proliferation as observed in normal cholangiocytes. Also, the stimulation of α_2_-AR and GABA receptors (using cAMP as a second messenger) seems to inhibit CCA growth, suggesting possible anticancer activity. Finally, TGR5, another G-protein-coupled receptor, is constitutively overexpressed in CCA, where it sustains tumor expansion and diffusion with a mechanism partially involving cAMP and different from what is observed in normal cholangiocytes. These and other findings suggest that the study of cAMP signaling in CCA may be useful to understand the differences between normal and neoplastic biliary cells and help to design strategies to abolish or mitigate cancer growth with systemic therapy.

## Figures and Tables

**Table 1:** Main preclinical studies evaluating cAMP/PKA signaling in cholangiocarcinoma (CCA).

Author Reference	Target	Experimental Setting	cAMP Levels	Results
Tan, C.K. [91]	Stimulation of somatostatin receptor (SSTR2)	CCA cell culture Human CCA specimens CCA xenotransplantation in nude mice	Unchanged	Reduced cancer growth
Kanno, N. [92]	Stimulation of α2-adrenergic receptor	CCA cell culture	Increased	Reduced cancer growth
Fava, G. [93]	Stimulation of γ-aminobutyric acid (GABA) receptor	CCA cell culture CCA xenotransplantation in nude mice	Increased	Reduced cancer growth and spread
Onori, P. [90]	Stimulation of secretin receptor	CCA cell culture Human CCA specimens CCA xenotransplantation in nude mice	Unchanged	Reduced cancer growth
Reich, M. [94]	Stimulation of Takeda G-protein-coupled receptor 5 (TGR5), receptor	CCA cell culture CCA xenotransplantation in nude mice	Increased	Enhanced cancer growth, reduced apoptosis
Loilome, W. [97]	PKA subunit RIα suppression	RIα knockdown in CCA cell culture	Not assessed	Reduced cancer growth
Panya, A. [98]	PKA subunit RIα suppression	T-cells against RIα subunit in CCA cell culture	Not assessed	Reduced cancer growth
